# Attenuated platelet aggregation in patients with septic shock is independent from the activity state of myosin light chain phosphorylation or a reduction in Rho kinase-dependent inhibition of myosin light chain phosphatase

**DOI:** 10.1186/s40635-014-0037-7

**Published:** 2015-02-12

**Authors:** Benjamin AJ Reddi, Samantha M Iannella, Stephanie N O’Connor, Adam M Deane, Scott R Willoughby, David P Wilson

**Affiliations:** Intensive Care Unit, Royal Adelaide Hospital, North Terrace, Adelaide, South Australia 5000 Australia; Molecular Physiology of Vascular Function Laboratory, School of Medical Sciences, University of Adelaide, Adelaide, South Australia 5000 Australia; Discipline of Acute Care Medicine, University of Adelaide, Adelaide, South Australia 5000 Australia; Centre for Heart Rhythm Disorders, School of Medicine, University of Adelaide, Adelaide, South Australia 5000 Australia

**Keywords:** Platelets, Sepsis, Shock, Aggregation, Rho kinase, Myosin phosphatase, Whole blood impedance aggregometry, Illness severity score

## Abstract

**Background:**

Impaired coagulation contributes to the morbidity and mortality associated with septic shock. Whether abnormal platelet contraction adds to the bleeding tendency is unknown. Platelets contract when Ca^2+^-dependent myosin light chain kinase (MLCK) phosphorylates Ser19 of myosin light chain (MLC_20_), promoting actin-myosin cross-bridge cycling. Contraction is opposed when myosin light chain phosphatase (MLCP) dephosphorylates MLC_20_. It is thought that Rho kinase (ROK) inhibits MLCP by phosphorylating Thr855 of the regulatory subunit MYPT, favouring platelet contraction. This study tested the hypotheses that in septic shock, (i) platelet function is inversely correlated with illness severity and (ii) ROK-dependent MLCP inhibition and myosin light chain phosphorylation are reduced.

**Methods:**

Blood was sampled from non-septic shock patients and patients in the first 24 h of septic shock. Platelet function was assessed using whole blood impedance aggregation induced by 1) ADP (1.6 and 6.5 μM), 2) thrombin receptor-activating protein (TRAP; 32 μM), 3) arachidonic acid (500 μM) and 4) collagen (3.2 μg/ml). Arachidonic acid-induced aggregation was measured in the presence of the ROK inhibitor Y27632. Illness severity was evaluated using sequential organ failure assessment (SOFA) and acute physiology and chronic health evaluation (APACHE) II scores. Western blot analysis of [Ser19]MLC_20_ and [Thr855]MYPT phosphorylation quantified activation and inhibition of platelet MLC_20_ and MLCP, respectively. Data were analysed using Spearman's rank correlation coefficient, Student's *t*-test and Mann-Whitney test; *p* < 0.05 was considered significant.

**Results:**

Agonist-induced aggregation was attenuated in septic shock patients (*n* = 22 to 34; *p* < 0.05). Aggregation correlated inversely with SOFA and APACHE II scores (*n* = 34; *p* < 0.05). Thr855 phosphorylation of MYPT from unstimulated platelets was not decreased in patients with septic shock (*n* = 22 to 24). Both septic shock and ROK inhibition attenuated arachidonic acid-induced platelet aggregation independent of changes in [Ser19]MLC_20_ and [Thr855]MYPT phosphorylation (*n* = 14).

**Conclusions:**

Impairment of whole blood aggregation in patients within the first 24 h of septic shock was correlated with SOFA and APACHE II scores. Attenuated aggregation was independent of molecular evidence of diminished platelet contraction or reduced ROK inhibition of MLCP. Efforts to restore platelet function in septic shock should therefore focus on platelet adhesion and degranulation.

## Background

Septic shock is fatal in over 20% of cases in the developed world [1,2]. Coagulation abnormalities are common in septic shock, ranging from mild thrombocytopaenia to disseminated intravascular coagulation. Whilst thrombocytopaenia is established as occurring frequently and of prognostic importance in sepsis [3], the nature of qualitative changes in platelet function remains incompletely understood.

Analysis of platelet function in the context of infection and sepsis has yielded equivocal data depending on the experimental paradigm. When platelets isolated from healthy volunteers are exposed to bacterial lipopolysaccharide (LPS) [4-7] or lipoteichoic acid (LTA) [8], or sepsis-associated cytokines [9], aggregation has been shown to be enhanced, inhibited or unchanged. In contrast, platelets isolated from septic shock patients show a profound and consistent reduction in adhesion and aggregation response to a range of agonists [10-13], despite biochemical evidence of increased platelet activity [14,15]. This suggests an intrinsic abnormality of a common effector pathway - adhesion, secretion or contraction - that persists even when the platelet is removed from the septic milieu. Considerable effort has investigated the role of abnormal platelet adhesion; however, the integrity of platelet contraction has not been specifically assessed in the context of sepsis or septic shock.

**Figure 1:**
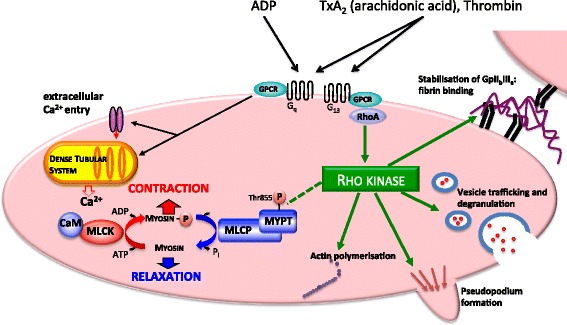
**Platelet aggregation requires contraction, adhesion and secretion.** ROK promotes platelet aggregation independent of MLCP inhibition. Platelet contraction requires Ser19 myosin light chain (MLC_20_) phosphorylation by Ca^2+^:calmodulin (CaM)-dependent myosin light chain kinase (MLCK). Agonists such as arachidonic acid initiate platelet contraction by augmenting [Ca^2+^]_cyt_ derived from both the extracellular fluid via transient receptor potential (TRP) channels and intracellular dense tubular system stores. Contraction is opposed when MLC_20_ is dephosphorylated by myosin light chain phosphatase (MLCP). It is proposed that MLCP is regulated by two mechanisms: (i) G protein-coupled receptors (GPCRs) bearing the Gα_13_ subunit activate membrane-bound RhoA, which activates Rho kinase (ROK) [23]; ROK is thought to inhibit MLCP by phosphorylating Thr855 on MYPT, the regulatory subunit of MLCP [24-26] (dashed line). (ii) PKC-dependent CPI-17 directly inhibits MLCP [24,27-29] (not shown). By inhibiting MLCP, both ROK and CPI-17 favour contraction independent of the prevailing [Ca^2+^]. Other than disinhibiting MLCP, there are other mechanisms by which ROK inhibition might oppose platelet aggregation: ROK is also involved in vesicle trafficking and degranulation, pseudopodium formation, stabilisation of platelet-fibrin binding and actin polymerisation.

Activated platelets contract with similar force to muscle - by making the clot more rigid and adherent, the platelet maintains clot shape and promotes uniform retraction [16]. Platelet contraction is determined by two opposing enzymatic processes (illustrated in Figure 1): (i) myosin light chain kinase (MLCK) phosphorylates Ser19 of myosin regulatory light chain (MLC_20_) [17,18] to promote actin-myosin cross-bridge cycling and contraction [19]; (ii) myosin light chain phosphatase (MLCP) dephosphorylates [Ser19]MLC_20_, uncoupling actin and myosin and favouring relaxation [20]. MLCK is stimulated by increased cytosolic Ca^2+^ derived either from the extracellular space [21] or from intracellular Ca^2+^ stores [22]. MLCP has been reported to be regulated by two mechanisms: (i) RhoA activates Rho kinase (ROK) [23] which inhibits MLCP by phosphorylating [Thr855]MYPT, the regulatory subunit of MLCP [24-26]. (ii) Activated PKC phosphorylates [Thr38]CPI-17 which directly and specifically inhibits MLCP [24,27-29]. By inhibiting MLCP, both RhoA/ROK and PKC/CPI-17 activities favour contraction, potentially explaining how platelets can be stimulated to change shape in the absence of significant changes in cytosolic [Ca^2+^]_in_ [30,31] and why aggregation is attenuated in platelets lacking the Gα_13_ (RhoA activating) subunit [32].

Previous studies have evaluated aggregation of platelets from patients with sepsis; however, our analysis of platelet function was restricted to patients with septic shock. Since septic shock is characterised by uncontrolled vasodilation and recognising that vascular smooth muscle and platelets may share common contractile signalling pathways [33,34], we explored whether a common mechanism might compromise both vascular smooth muscle contraction and platelet contraction. Contractile dysfunction in vascular smooth muscle has been associated with increased MLCP activity in animal models of sepsis. In particular, inhibitory [Thr855] phosphorylation of MYPT (the target of RhoA/ROK) is reduced in both an endotoxaemic rat model [35] and a caecal ligation and puncture mouse model [36].

Both whole blood impedance aggregometry and Western blot analysis of P-[Ser19]MLC_20_ and the ROK substrate P-[Thr855]-MYPT were performed to test the following hypotheses: 1) whole blood aggregation of platelets is attenuated in patients with septic shock, 2) platelet aggregation in septic shock patients correlates with illness severity indexed by the SOFA and APACHE II scores, and 3) platelet aggregation in septic shock patients is associated with reduced (i) [Ser19]MLC_20_ phosphorylation and (ii) RhoA/ROK-mediated inhibition (Thr855 phosphorylation) of myofilament-associated MLCP.

The integrity of platelet contraction and its potential as a therapeutic target is an important gap in our understanding of the pathogenesis of platelet dysfunction in human septic shock.

## Methods

### Patients

This prospective observational study was approved by the research ethics committees of the University of Adelaide and the Royal Adelaide Hospital. Consecutively identified patients aged 18 to 80 years admitted into either the elective surgery unit (controls) or specifically the intensive care unit of the Royal Adelaide Hospital on randomly allocated sampling days between September 2012 and May 2013 meeting the eligibility criteria were enrolled into the study; 40 patients were recruited with acute (<24 h) septic shock, and 40 patients were recruited into the non-septic shock control group. The diagnosis of septic shock was defined as the presence of two or more of the following: (i) temp >38°C or <36°C, (ii) heart rate >90 bpm, (iii) respiratory rate >20 or PaCO_2_ <32 mmHg, (iv) leucocyte count >12,000 or <4,000/mm^3^ or >10% bands, *plus* suspected or proven source of infection, organ dysfunction attributable to sepsis and hypotension/requirement for vasopressor support >1 h despite adequate fluid resuscitation. Patients were managed according to current international guidelines [37]. These diagnostic criteria were absent in control patients who were post-operative elective surgical patients in whom an indwelling arterial catheter had been sited for routine intra-operative monitoring. Because agonist-induced platelet aggregation has been shown to be enhanced beyond 24 h after surgery [38], samples were drawn a maximum of 8 to 12 h post surgery. Exclusion criteria included co-incident malignancy, pre-existing platelet abnormality, patients receiving anti-platelet medications or platelet transfusions over the last 28 days, patients undergoing extracorporeal membrane oxygenation or admission within the last 72 h with severe trauma. Patients with platelet count <150,000/μl (measured max 4 h before sampling or at the time of sampling) were excluded from functional analysis.

Blood samples were taken and demographic and clinical data were recorded from all patients enrolled in the study after written informed consent was acquired from patients or their next of kin.

### Illness severity

Illness severity was evaluated using two different composite scoring tools: the sequential organ failure assessment (SOFA) and acute physiology and chronic health evaluation (APACHE) II scores. The former incorporates respiratory, cardiovascular, neurological, liver, renal and coagulation (including platelet count) parameters [39]; the latter incorporates age, previous health status and 12 acute cardiovascular, respiratory, neurological, haematological and renal parameters (notably platelet count is not included) [40].

### Sample preparation

A volume of 12 ml of blood was drawn from an existing arterial line into 3.2% sodium citrate tubes (VACUETTE®, Greiner Bio-One, Kremsmünster, Austria). Samples were partitioned to be analysed either by whole blood impedance aggregometry or to undergo protein extraction for Western blot analysis of the phosphorylation state of [Ser19]-MLC_20_ and [Thr855]-MYPT.

### Platelet aggregation

Whole blood impedance aggregometry (Multiplate® aggregometer, Dynabyte, Munich, Germany) was performed within 1 h of blood collection according to the manufacturer's instruction. Samples were not normalised by platelet concentration since Multiplate whole blood impedance aggregometry by the Multiplate system has been shown to be relatively independent of platelet concentration when the count is >150,000/μl [41]. Citrated whole blood (300 μl) was diluted with physiological saline (300 μl; 37°C) followed by incubation for 3 min at 37°C. Following administration of agonists, platelet aggregation (measured in aggregation units (AU)) was recorded for 6 min. Aggregation recording was repeated if the area under the curve detected by each of the two electrodes varied more than 20% from the mean. As a further control to exclude the influence of platelet count on aggregation response, results of aggregation induced by adenosine diphosphate (ADP; 6.5 μM) and arachidonic acid (500 μM) were plotted against platelet count for samples from both septic shock and non-septic shock patients; no correlation was found (*n* = 34, *p* > 0.05; data not shown).

In order to ascertain whether there was a generalised impairment of platelet aggregation that was consistent with a reduction in [Ser19]MLC_20_ and [Thr855]MYPT phosphorylation-dependent contractility, the aggregation response to agonists was measured: thrombin receptor-activating protein (TRAP; 32 μM) and arachidonic acid (500 μM), both associated with RhoA/ROK-mediated inhibition of MLCP, and ADP (1.6 and 6.5 μM) and collagen (3.2 μg/ml), which are RhoA/ROK independent [aggregation agonists purchased were sourced from Multiplate®] [42].

In order to identify whether platelet aggregation was impaired by inhibition of the RhoA/Rho kinase pathway, the aggregation response to arachidonic acid was also measured in the presence or absence of 20 min of incubation with the Rho kinase inhibitor Y27632 (1 μM) [(1R,4r)-4-((R)-1-aminoethyl)-N-(pyridin-4-yl)cyclohexanecarboxamide, Calbiochem-Novabiochem Corp., San Diego, CA, USA] and Y27632 alone. Additional controls included aggregation responses of platelets incubated in buffer alone or Y27632 alone.

### Western blot analysis

To identify whether the reduction in aggregation of platelets from patients suffering from septic shock was associated with reduced activity of the contractile proteins, Western blot analyses were used to measure the state of MLC_20_ phosphorylation (P-[Ser19]MLC_20_) in 1) unstimulated platelets, 2) arachidonic acid-stimulated platelets, 3) arachidonic acid-stimulated platelets pre-incubated with Y27632 for 20 min and 4) platelets incubated with Y27632 only. To address whether the P-[Ser19]-MLC_20_ was associated with altered RhoA/ROK-dependent Ca^2+^ sensitivity, the Thr-855 phosphorylation state of the MYPT P-[Thr855]MYPT targeting subunit of MLCP was directly measured in the same platelets. Results were compared for platelets from both non-septic and septic shock patients.

Since no increase in Thr855 phosphorylation of platelet MYPT has been specifically identified in the literature, we used a comparison of unstimulated and TxA_2_ receptor agonist (U46619)-stimulated rat mesenteric artery as a positive control [24]; rat caudal artery samples were transferred with the platelet samples.

Briefly, platelet-rich plasma was isolated by centrifuging 2 ml of whole blood at 800 rpm (in an Eppendorf microfuge) for 12 min and decanted. The platelet-rich plasma was then stimulated with arachidonic acid (500 μM), Y27632 (1 μM) or both before being centrifuged at 2,600 rpm for 18 min to yield a platelet-rich pellet. The plasma was discarded and the platelet-rich pellet washed three times in phosphate-buffered saline. Enzymatic activity in platelets was stopped by incubating for 30 s in a solution of ice-cold 10% trichloroacetic acid (TCA)/acetone. The precipitated protein was rinsed three times with ice-cold acetone to remove residual TCA.

A washed platelet-rich pellet from 2 ml of whole blood was solubilised in 200 μl glycerol-free sample buffer containing 50 mM Tris-HCl, pH 6.8, 1% (*w*/*v*) SDS, 1× cOmplete™ protease inhibitor cocktail (Roche, Basel, Switzerland), 100 μM di-isopropylfluorophosphate (Sigma-Aldrich, Castle Hill, Australia), 10 mM DTT, 20% (*w*/*v*) sucrose and 0.1% (*w*/*v*) bromophenol blue. Samples were heated to 95°C for 5 min and then vortex mixed for 90 s. Samples were centrifuged in a microfuge at 12,000 rpm for 4 min to remove insoluble particulates prior to SDS/PAGE using Bio-Rad 4–15% Criterion TGX precast gels run in a Bio-Rad Criterion unit at 200 V for 60 min (Bio-Rad, Hercules, CA, USA). Gels were stained with Coomassie Brilliant Blue R-250 (Merck and Co., Macquarie Park, Australia) and scanned using the LI-COR Odyssey system (LI-COR Biotechnology, Lincoln, NE, USA) to quantify the total *extracted* protein and allow normalisation of total protein content in the samples. Following normalisation of extracted protein content in samples, SDS/PAGE was repeated and proteins were electrophoretically transferred onto 0.22 μm nitrocellulose (Bio-Rad) (100 V for 30 min) in transfer buffer containing 25 mM Tris/HCl, 192 mM glycine, 20% (*v*/*v*) methanol and 0.1% (*w*/*v*) SDS for MYPT and without SDS for MLC_20_. Following protein transfer to nitrocellulose, non-specific binding sites were blocked with a solution containing 50% LI-COR Odyssey™ blocking buffer/50% Tris-buffered saline (TBS; 50 mM Tris, pH 7.4, 150 mM NaCl) for 60 min, followed by incubation with TBS plus 0.05 Tween-20 (TBS-T) containing either mouse anti-MYPT1 (1:1,000) [made in-house [24]], rabbit anti-P-[Thr855]MYPT1 (1:1,000) [Upstate/Millipore USA, Inc., Billerica, MA, USA], mouse anti-MLC_20_ [Santa Cruz Biotechnology, Santa Cruz, CA, USA] or mouse anti-P-[Ser19]MLC_20_ (1:1,000). The nitrocellulose was washed three times in TBS-T and incubated in TBS-T with a 1 to 10,000 dilution of biotin-conjugated goat anti-mouse or goat anti-rabbit secondary antibody [Pierce/Thermo Scientific Australia, Scoresby, Australia] for 60 min before another three washes with TBS-T. The nitrocellulose was then incubated for 60 min in TBS-T with a 1 to 10,000 dilution of streptavidin conjugated to the 800-nm DyLight fluorochrome [Pierce/Thermo Scientific Australia]. Fluorescence was detected and quantified using the LI-COR Odyssey™ system. All samples were transferred, incubated and scanned together to ensure identical analysis conditions between septic shock and non-septic shock patients and between different platelet treatments. All samples used in the analysis were within the linear range of detection.

### Data analysis

Western blot data was expressed as a ratio of total P-[Thr855]/total MYPT and total P[Ser19]-MLC_20_/total MLC_20_. Sample size was determined by a power analysis based upon data derived from Western blot analysis of MYPT phosphorylation in vascular smooth muscle [24]. Statistical analysis was performed using GraphPad Prism v. 6 (GraphPad Software, Inc., La Jolla, CA, USA). Data were tested with the D'Agostino and Pearson omnibus normality test. All results are presented as the mean ± SEM where *n* indicates the number of independent experiments for each treatment. For parametric and non-parametric data, unpaired Student's *t*-tests and Mann-Whitney tests, respectively, were used to compare data between groups. For parametric and non-parametric data, paired Student's *t*-test and Wilcoxon test, respectively, were used to compare within-patient agonist/inhibitor responses; *p* < 0.05 was considered statistically significant. Asterisks indicate statistically significant differences from control. Spearman's rank-order correlation coefficient was calculated for the relationships between (i) noradrenaline dose and platelet aggregation response to arachidonic acid and ADP, (ii) noradrenaline dose required to achieve the desired mean arterial pressure and Thr[855] phosphorylation state of MYPT, (iii) SOFA and APACHE II scores and aggregation responses to arachidonic acid and ADP and (iv) SOFA and APACHE II scores and the Thr[855] phosphorylation state of MYPT. A correlation between TRAP/collagen-induced platelet aggregation and illness severity scores was not performed as the number of samples was too small. Fisher's test was used to analyse the mortality difference between the groups. A mixed-effects regression analysis was undertaken (Stata/MP 13.1) to identify significant interactions between variables and to estimate the overall effect of septic shock on platelet aggregation.

## Results

Patients with septic shock (37) and non-septic shock controls (40) were recruited and were well matched for demographic characteristics; clinical status at the time of blood sampling was recorded and represented in Table 1 with clinical outcome data. The mean platelet count was in the normal range for both septic shock (mean 278; SEM 24.27) and non-septic shock (mean 261; SEM 12.44) patients, consistent with the early stage of septic shock at which whole blood was acquired [43]. Hospital and ICU mortality was higher (*p* < 0.01) in septic shock patients.

**Table 1:** **Clinical status at the time of blood sampling**

	**Septic shock**	**Non-septic shock**
Number	37	40
Age	60.54 ± 13.55	60.5 ± 14.52
Female, *n* (%)	26 (70)	27 (68)
Smoker, *n* (%)	12 (32)	8 (20)
APACHE II score^a^	24.14 (1.19)	NA
SOFA score^b^	9.5 (0.47)	NA
Proven infection, *n* (%)	26 (70)	NA
Gram-positive	8	NA
Gram-negative	10	NA
Mixed	5	NA
Fungal	3	NA
Mean catecholamine dose (μg/min)	18.7 (2.03)	NA
Platelet count (10^9^/l)	278 (24.3)	261 (12.4)
ICU mortality (%)	10 (27)	0
Hospital mortality, *n* (%)	16 (43)	2 (5)

Unless otherwise stated, values indicate mean and SEM; *p* > 0.05 for all comparisons except ICU and hospital mortality, where *p* < 0.01. ^a^Acute physiology and chronic health evaluation (APACHE) II score, a higher value indicates greater admission illness severity. ^b^Sequential organ failure assessment (SOFA) scores range from 0 to 24 with higher scores indicating more severe organ dysfunction. NA, non-applicable.

**Figure 2:**
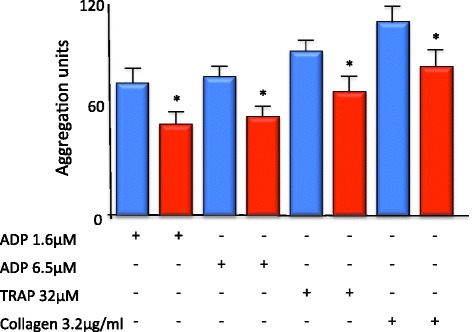
**Whole blood platelet aggregation is reduced in patients with septic shock.** Whole blood platelet aggregation in response to ADP, thrombin receptor-activating protein (TRAP) and collagen was attenuated in patients suffering from septic shock (red bar) vs. non-septic shock controls (blue bar). (*n* = 16 to 20 per patient group, *p < 0.05).

Platelet aggregation in response to each agonist was significantly attenuated in patients with septic shock. Compared with platelets from non-septic shock patients, platelets from patients with septic shock demonstrated attenuated whole blood impedance aggregation in response to low-dose ADP (74.3 ± 8.7 vs. 51.2 ± 6.7), high-dose ADP (78.1 ± 7.5 vs. 55.5 ± 6.8), thrombin receptor-activating protein (92.4 ± 6.8 vs. 69.3 ± 9.4) and collagen (108.8 ± 9.4 vs. 83.3 ± 11.1) (*n* = 16 to 20 per patient group, *p* < 0.05) (Figure 2). Regression analysis across agonists was used to model the overall effect of septic shock on aggregation; the overall estimate of the impact of septic shock on aggregation is −32% (−7.8, −55.7, *p* < 0.05).

Platelet aggregation was strongly promoted by arachidonic acid. Arachidonic acid-mediated platelet aggregation was attenuated in whole blood (i) taken from patients with septic shock and (ii) incubated with the Rho kinase inhibitor Y27632.

**Figure 3:**
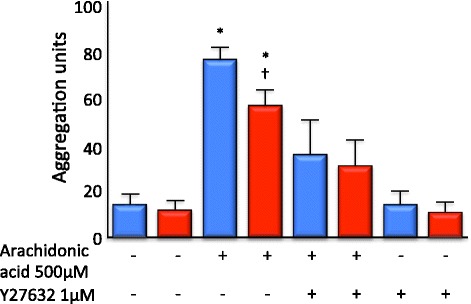
**Arachidonic acid-dependent platelet aggregation was reduced in patients with septic shock.** Platelet number was not significantly different between septic shock and non-septic shock patients; however, arachidonic acid-induced aggregation was reduced in platelets derived from septic shock patients (red bar) compared with non-septic shock controls (blue bar) (*n* = 26 to 33 per patient group, *p* < 0.05). Incubation for 20 min with the ROK inhibitor Y27632 significantly attenuated arachidonic acid-mediated platelet aggregation in whole blood from both septic shock and non-septic shock patients. Arachidonic acid-induced aggregation in the presence of Y27632 was not significantly different from aggregation of unstimulated platelets. (*n* = 10 to 12 per patient group, * and † *p* < 0.05).

Arachidonic acid (500 μM)-mediated platelet aggregation was attenuated in whole blood taken from patients with septic shock (76.9 ± 6.4 vs. 57.1 ± 7.5; *n* = 26 to 33, *p* < 0.05). To illustrate the proof of principle that ROK inhibition can attenuate platelet function, platelets were incubated for 20 min with the ROK inhibitor Y27632. Incubation with Y27632 significantly attenuated arachidonic acid-mediated aggregation in whole blood from both non-septic shock and septic shock patients (35.3.0 ± 15.4 and 31.3 ± 11.3; *n* = 10 to 12, *p* < 0.05.) In the presence of Y27632, arachidonic acid-induced aggregation was not significantly different from aggregation of unstimulated platelets (13.5 ± 7.1 vs. 15.03 ± 5.0 (non-septic shock) and 11.7 ± 4.8 vs. 11.4 ± 3.6 (septic shock); *n* = 10 to 12) (Figure 3). Although regression modelling identified a positive interaction between ROK inhibition and septic shock supporting that the effect of ROK inhibition is lessened in the presence of septic shock, this was non-significant (*p* = 0.312).

The regression analysis was repeated controlling for platelet count, the result of which was consistent with our more simplified analysis and did not change our inference with respect to the effect of septic shock or ROK inhibition, namely the conclusion that septic shock was associated with a reduction in platelet aggregation in response to each of four agonists.

**Figure 4:**
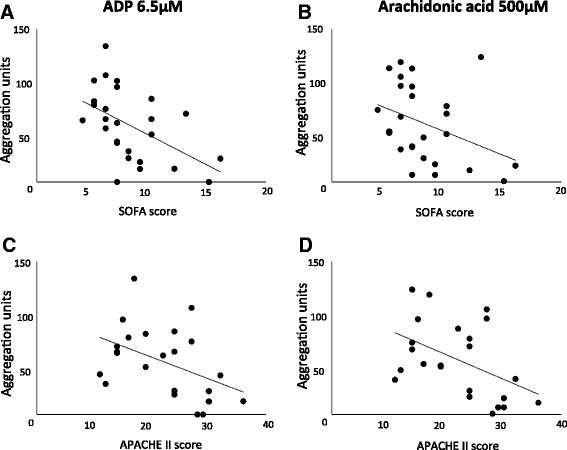
**Platelet aggregation is inversely correlated with illness severity.** There was a significant correlation (*p* < 0.05) between sequential organ failure assessment (SOFA) and acute physiology and chronic health evaluation (APACHE) II scores at the time of blood sampling and platelet aggregation induced by **(A, C)** ADP (*r* = −0.51 and −0.36) and **(B, D)** arachidonic acid (*r* = −0.41 and −0.47) in whole blood taken from patients with septic shock (*n* = 26). Note: platelet concentration was not significantly lower in the septic shock patients, and there was no correlation between platelet count and either SOFA or APACHE II score (*p* > 0.05).

Poor platelet aggregation responses were associated with high SOFA and APACHE II scores. Amongst patients with septic shock, the aggregation response to both ADP (6.5 μM) and arachidonic acid (500 μM) was inversely correlated with their SOFA score (*n* = 26, *r* = −0.51 and −0.36, respectively, *p* < 0.05; Figure 4A,B). Although the SOFA score includes platelet count, there was no correlation between platelet count and SOFA score in septic shock patients in this study and platelet count was similar in both septic shock and non-septic shock patients (Table 1). Furthermore, ADP- and arachidonic acid-induced aggregation correlated inversely with the SOFA score even when coagulation parameters were excluded (*p* < 0.05). Furthermore, ADP- and arachidonic acid-induced aggregation of platelets from septic shock patients was also inversely correlated with their APACHE II score (*n* = 26, *r* =−0.44 and −0.47, respectively, *p* < 0.05; Figure 4C,D); the APACHE II calculation does not include platelet count. Platelet aggregation was not significantly correlated with noradrenaline dose or mean arterial blood pressure (data not shown).

Contraction-associated P-[Ser19]MLC_20_ was increased in platelets stimulated with arachidonic acid; this increase was not attenuated in platelets from septic shock patients or by pre-incubation with Y27632.

**Figure 5:**
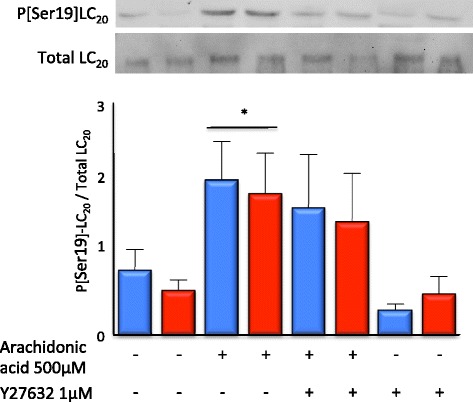
**Neither septic shock nor ROK inhibition reduced arachidonic acid-induced LC**_**20**_ **phosphorylation.** Following stimulation with arachidonic acid, Ser19 phosphorylation of LC_20_ was significantly increased in platelets from both septic shock (red bar) and non-septic shock control (blue bar) patients. Pre-incubation with Y27632 did not significantly attenuate the arachidonic acid-induced increase in Ser19 phosphorylation of LC_20_. Arachidonic acid-induced Ser19 phosphorylation of LC_20_ was not different in platelets derived from patients with septic shock compared to those derived from non-septic shock controls. (*n* = 14 to 15 per patient group, *p < 0.05).

Baseline P-[Ser19]MLC_20_ in unstimulated platelets was not different in platelets from patients with or without septic shock (0.84 ± 0.27 vs. 0.57 ± 0.14). Following stimulation with arachidonic acid, P-[Ser19]MLC_20_ was significantly higher in platelets from both non-septic shock and septic shock patients (2.0 ± 0.49 and 1.8 ± 0.53; *p* < 0.05), but the increase was not significantly different between non-septic shock and septic shock patients. Pre-incubation with Y27632 did not reduce the arachidonic acid-induced increase in P-[Ser19]MLC_20_ in platelets from either patient group (non-septic shock 1.5 ± 0.69 and septic shock 1.5 ± 0.63). The proportion of P-[Ser19]MLC_20_ in unstimulated platelets was not significantly reduced following pre-incubation with Y27632 (non-septic shock 0.31 ± 0.08 and septic shock 0.52 ± 0.23) (*n* = 14 per patient group; Figure 5).

The baseline Thr855 phosphorylation state of MYPT was not reduced in platelets taken from patients with septic shock.

**Figure 6:**
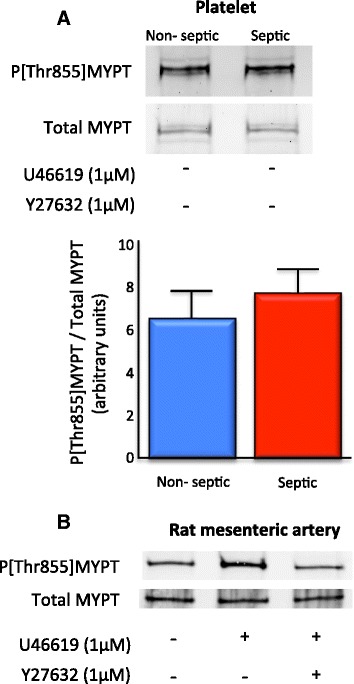
**ROK-dependent myosin phosphatase activity was unchanged in platelets isolated from patients with septic shock. (A)** The proportion of the myosin phosphatase regulatory subunit (MYPT) phosphorylated at the ROK substrate site Thr855 did not differ between unstimulated platelets taken from patients with septic shock compared to those taken from non-septic shock controls (*n* = 22 to 24 in each patient group). **(B)** Rat mesenteric artery samples were transferred with platelet samples as controls. Treatment with the stable thromboxane A_2_ mimetic U46619 increased ROK-dependent Thr855 phosphorylation of MYPT that was attenuated with the ROK antagonist Y-27632; representative data from *n* = 3.

The fraction of the MLCP regulatory subunit MYPT which was phosphorylated at the inhibitory Thr855 site (i.e. the proportion of MLCP in the putative RhoA/ROK-dependent inactive state) was not different in unstimulated platelets taken from either non-septic shock or septic shock patients (6.482 ± 1.32 vs. 7.658 ± 1.07; *n* = 22 to 24 per patient group; Figure 6). The Thr855 phosphorylation state of MYPT was not correlated with the noradrenaline dose, mean arterial pressure or SOFA/APACHE II scores (data not shown). Control experiments using rat mesenteric artery samples transferred alongside platelet samples identified that treatment with U46619 (1 μM), a mimetic of the arachidonic acid metabolite thromboxane A_2_, increased P[Thr855]MYPT (4.79 ± 1.27 vs. 14.71 ± 2.02) and incubation with Y-27632 (1 μM) attenuated U46619-mediated P[Thr855]MYPT.

In platelets from both septic shock and non-septic shock patients, the Thr855 phosphorylation state of MYPT was not significantly different in platelets stimulated with arachidonic acid and platelets stimulated with arachidonic acid following incubation with Y27632.

**Figure 7:**
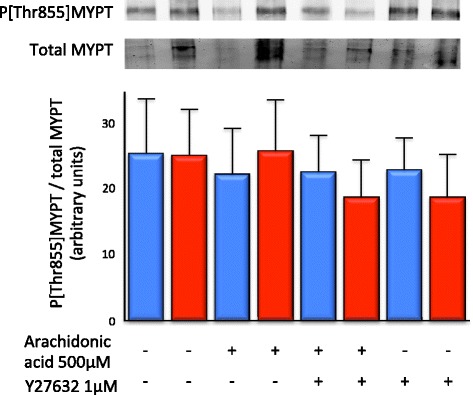
**Thr855 MYPT phosphorylation was not significantly influenced by arachidonic acid, ROK inhibition or septic shock.** The proportion of the ROK substrate MYPT phosphorylated at the inhibitory Thr855 site was not increased following stimulation with arachidonic acid and/or incubation with Y27632 and did not differ between samples from patients with septic shock (red bar) compared to samples from non-septic shock patients (blue bar) (*n* = 13 to 15 per patient group).

There was no significant increase in the Thr855 phosphorylation state of MYPT in platelets taken from either patient group following stimulation with arachidonic acid (non-septic shock 25.64 ± 8.16 vs. 22.05 ± 7.06; septic shock 25.27 ± 6.96 vs. 25.52 ± 8.02). There was no significant difference in the Thr855 phosphorylation state of MYPT in either group following stimulation with arachidonic acid of platelets that had been incubated with Y27632 (non-septic shock 22.25 ± 5.75 vs. septic shock 18.42 ± 5.91) (*n* = 13 to 15; Figure 7).

## Discussion

The global impact of septic shock is profound; in 2012, the Surviving Sepsis Campaign Guidelines Committee stated that severe sepsis and septic shock affect ‘millions of people around the world, killing one in four (and often more), and is increasing in incidence’ [37]. Up to 7% of patients with septic shock experience life-threatening haemorrhage [1]; although thrombocytopaenia contributes to haemorrhage in sepsis and is an independent risk factor for poor outcome [44], the presence, impact and mechanism of qualitative platelet dysfunction are less well established. The study tested the primary hypothesis that whole blood aggregation of platelets from patients with septic shock would be attenuated and that the degree of aggregation impairment would correlate with illness severity indexed by the SOFA and APACHE II scores. The secondary hypothesis that reduced platelet aggregation would be associated with (i) reduced activation of the myosin light chain, MLC_20_, and (ii) reduced RhoA/ROK-mediated inhibition of myofilament-associated MLCP was also tested.

Our data add to the weight of evidence that platelet function is impaired in sepsis [10,45], identifying for the first time that specifically in human septic shock, there is a clear reduction in ADP-, thrombin receptor-activating protein-, arachidonic acid- and collagen-dependent whole blood impedance aggregation.

Platelets are activated when sub-endothelial collagen and the von Willebrand factor (vWF) are exposed and bind to their respective platelet receptors [46,47]. This triggers the activation of integrins, which bind platelets to the extracellular matrix. Platelets then enter a secretion phase, emptying α-granules and dense granules which contain soluble mediators such as ADP [48]. Activated platelets also generate and secrete thromboxane A_2_ (TxA_2_) *de novo* [49]. ADP, TxA_2_ and locally produced thrombin [50] trigger platelet contraction through heterotrimeric G proteins of the G_q_ and G_13_ families [34].

Whole blood impedance aggregometry has advantages over optical-based techniques [51] since platelets (i) remain in the same humoral and cellular milieu as *in vivo*, (ii) do not suffer centrifugation injury, (iii) are analysed sooner after phlebotomy and (iv) are derived from smaller blood volumes. Whole blood impedance aggregometry thus permits assessment of platelet function in as close to *in vivo* conditions as possible. Generalised attenuation of aggregation to different agonists using independent receptor pathways suggests an abnormality downstream of receptor and second messenger mechanisms, indicating a failure of one or more of the following: adhesion, secretion or contraction.

The reduction in agonist-dependent aggregation in patients with septic shock correlated with higher SOFA (with and without inclusion of the coagulation score) and APACHE II scores even amongst septic shock patients with relatively preserved platelet counts. The finding that platelet function provides an early index of disease severity in patients with septic shock is not surprising since platelets are directly immersed in the milieu of inflammatory mediators and bacterial products. This finding warrants further study to identify whether using whole blood impedance aggregation improves prognostic accuracy in septic shock.

Platelet aggregation and clot formation depend upon platelet shape change, drawing the discoid resting platelet into a stiffer structure whilst extending pseudopodia to facilitate adhesion and clot stability; platelet contraction is critical for maintaining the primary haemostatic plug [52]. We postulated that diminished MLC_20_ phosphorylation, resulting from reduced ROK-dependent inhibition of MLCP, attenuated platelet contraction and hence aggregation in sepsis.

Aggregation of platelets from both non-septic and septic shock patients was profoundly attenuated in the presence of the ROK inhibitor Y27632, demonstrating that, in principle, if septic shock were associated with impaired ROK-mediated inhibition of MLCP, this could effectively inhibit platelet aggregation. However, contrary to our hypothesis, our data show that whilst both septic shock and incubation with a ROK inhibitor were associated with reduced whole blood aggregation, in neither case was the attenuation in aggregation associated with a significant reduction in [Thr855] phosphorylation of MYPT or [Ser19] phosphorylation of MLC_20_, i.e. attenuation of aggregation was independent of ROK-mediated inhibition of MLCP or activation of myosin (dashed line in Figure 1). These data provide two insights: Firstly, reduced whole blood aggregation in septic shock was independent of molecular evidence of reduced actin-myosin contraction (no change in myosin LC_20_ phosphorylation), instead suggesting impairment of other elements of platelet function such as adhesion or granule secretion. Consistent with our finding that reduced platelet aggregation in septic shock was not a consequence of reduced ROK-mediated Thr855 phosphorylation of MYPT was the observation that aggregation was also attenuated in response to collagen and ADP, agonists thought to activate platelets independent of Gα_12/13_ and RhoA/ROK [34].

Secondly, treatment with the ROK inhibitor Y27632 attenuated platelet aggregation independent of significant changes in the phosphorylation state of [Thr855]MYPT and phosphorylation of MLC_20_. There are several alternative mechanisms by which ROK inhibition might attenuate platelet aggregation independent of changes in MLC_20_ phosphorylation [53], and these are illustrated in Figure 1. Specifically, ROK also (i) maintains active binding of the integrin GPIIbIIIa to fibrin and vWF [54,55], and it has been identified that activation of GPIIbIIIa is decreased in patients with sepsis [13]; (ii) mediates vesicle trafficking within platelets independent of MLCP [56-59]; (iii) is essential for TxA_2_- and thrombin-induced granule secretion [32,60] and (iv) modulates actin assembly and polymerisation in the dynamic regulation of microtubule coils [61-63] and formation of stress fibres [64]. It is now clear that ROK inhibition may disrupt any of these mechanisms involved in platelet aggregation without significantly reducing the proportion of phosphorylated [Thr855]MYPT and [Ser19]MLC_20_.

Vascular smooth muscle contraction depends on Ca^2+^-mediated activation of MLCK and RhoA/ROK-mediated inhibition of MLCP. Animal studies suggest that diminished contraction of vascular smooth muscle in sepsis is associated with attenuated ROK activity and disinhibition of MLCP [35,36]. Furthermore, common bacterial pathogens produce toxins capable of inhibiting RhoA/ROK-mediated inhibitory phosphorylation of MLCP [65-67] (reviewed by Somlyo and Somlyo [27]). Whilst platelets also contain ROK, actin-myosin-dependent platelet contraction does not appear to be mediated by ROK inhibition of MLCP, and septic shock-induced platelet dysfunction does not appear to be a consequence of platelet contractile dysfunction. Our data suggest that in patients with septic shock, targeted support of platelet adhesion and secretion is likely to be of more value in restoring platelet function than targeting platelet contraction.

## Conclusions

Platelets in whole blood derived from patients suffering from septic shock exhibit reduced aggregation to a range of agonists, indicating an increased bleeding risk despite a normal platelet count. The reduction in aggregation correlated with illness severity scores. Therefore, reduced platelet aggregation in whole blood from patients with septic shock may be valuable as an early prognostic marker associated with poor patient outcome. Reduced platelet aggregation in patients with septic shock was independent of molecular evidence of reduced platelet contraction, i.e. [Ser19]MLC_20_ phosphorylation or RhoA/ROK-dependent inhibitory phosphorylation of [Thr855]MYPT. Future efforts to ameliorate coagulopathy in sepsis should consider mechanisms that include platelet adherence and secretion.
